# Supplementary material for: Splice-Junction-Based Mapping of Alternative Isoforms in the Human Proteome
Source: Cell Rep. Author manuscript; Available in PMC 2020 Jan 15. (PMC6961840; doi:10.1016/j.celrep.2019.11.026)
Supplement: 2 [file NIHMS1546469-supplement-2.zip › DF1/PXD006675/RightAtrium/RightAtrium_49_GCA_LSAQCYVAIFLEFGNFSIQV.pdf]

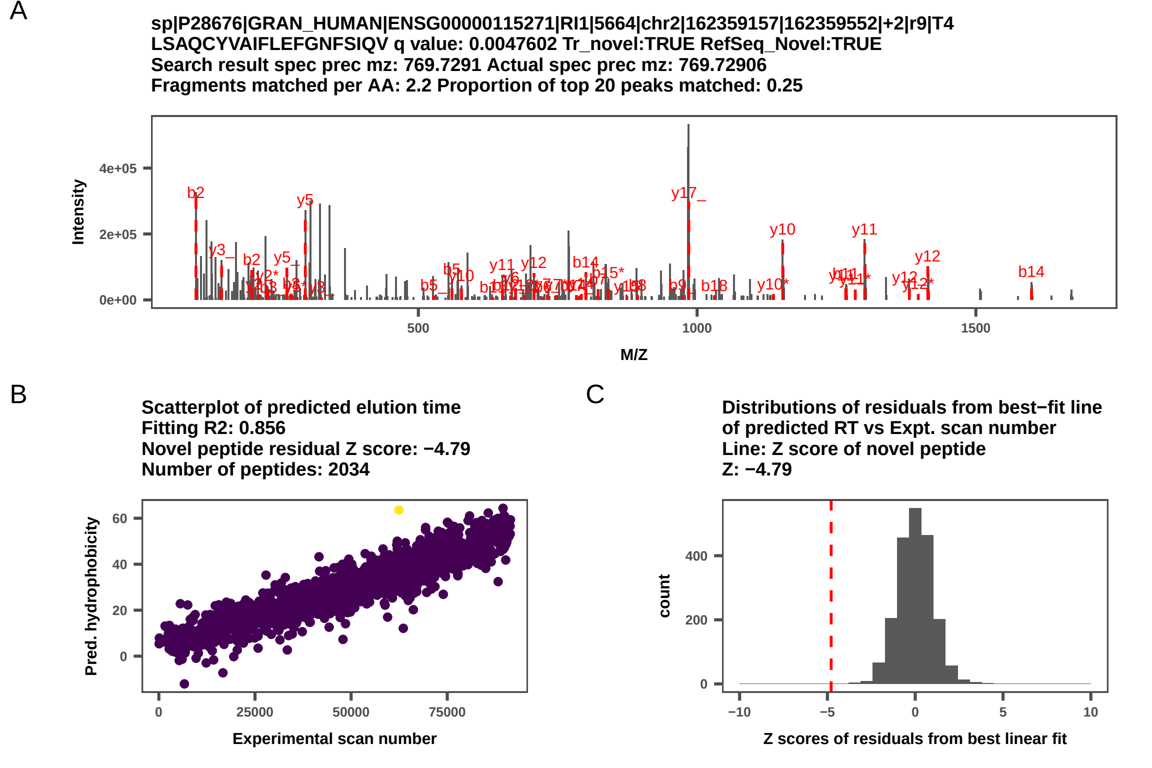
A
sp|P28676|GRAN_HUMAN|ENSG00000115271|RI1|5664|chr2|162359157|162359552|+2|r9|T4
LSAQCYVAIFLEFGNFSIQV q value: 0.0047602 Tr_novel:TRUE RefSeq_Novel:TRUE
Search result spec prec mz: 769.7291 Actual spec prec mz: 769.72906
Fragments matched per AA: 2.2 Proportion of top 20 peaks matched: 0.25
Intensity
4e+05
2e+05
0e+00
b2
y5
y17_
y10
y11
y12
b14
500
1000
1500
M/Z
B
Scatterplot of predicted elution time
Fitting R2: 0.856
Novel peptide residual Z score: −4.79
Number of peptides: 2034
Pred. hydrophobicity
60
40
20
0
0
25000
50000
75000
Experimental scan number
C
Distributions of residuals from best−fit line
of predicted RT vs Expt. scan number
Line: Z score of novel peptide
Z: −4.79
count
400
200
0
−10
−5
0
5
10
Z scores of residuals from best linear fit
